# Supplementary material for: ON/OFF domains shape receptive field structure in mouse visual cortex
Source: Nat Commun. 2022 May 5;13:2466. doi: 10.1038/s41467-022-29999-7 (PMC9072422; doi:10.1038/s41467-022-29999-7)
Supplement: Supplementary file 1 — Supplementary Information [file 41467_2022_29999_MOESM1_ESM.pdf]

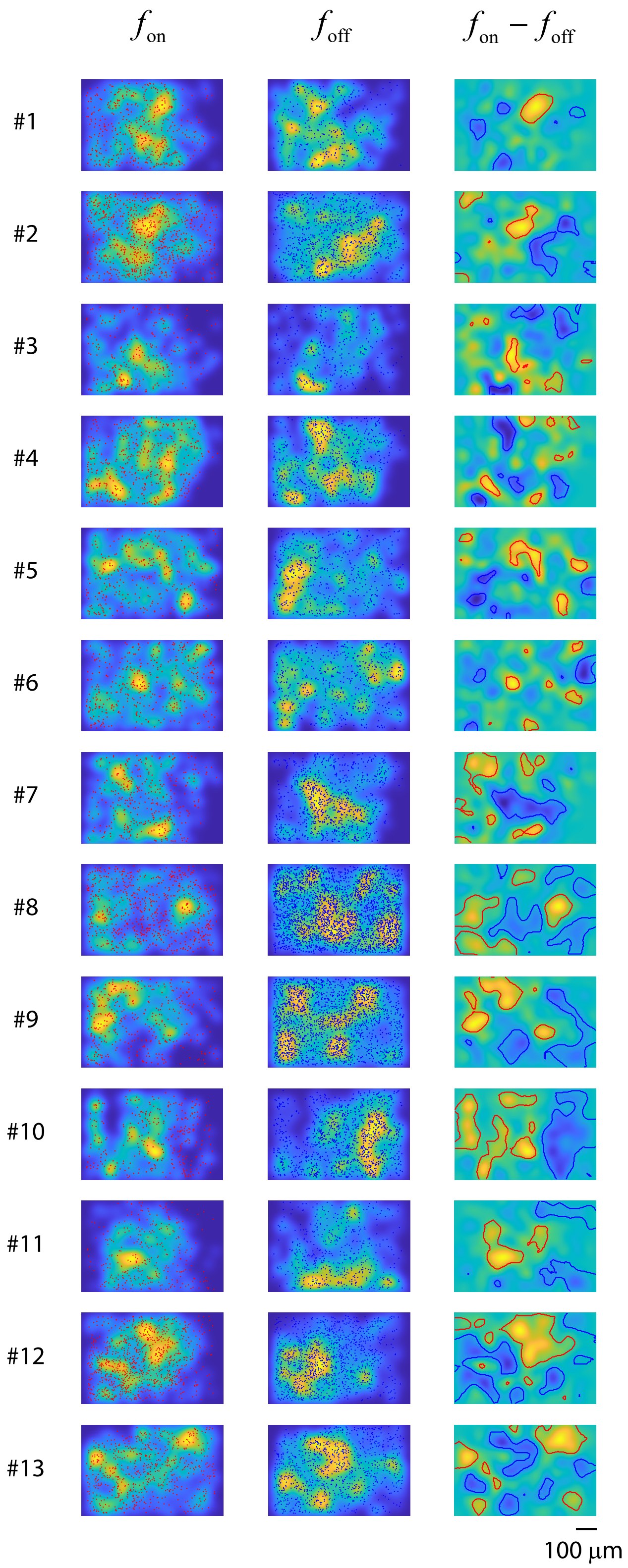

Supplementary Fig 1. Demonstration of ON/OFF domains in native cortical space. Each panel shows the result of one experimental dataset. In each case, the image on the left shows the distribution of ON cells on the cortical surface (ignoring depth) along with a pseudo-color map showing the estimated density. The density estimation for OFF cells appears in the middle panel. Level sets depict areas where the fluctuations exceed what might be expected by chance at a 0.001 level by randomly shuffling the ON/OFF labels of the cells (without changing their positions).
