# Supplementary material for: ON/OFF domains shape receptive field structure in mouse visual cortex
Source: Nat Commun. 2022 May 5;13:2466. doi: 10.1038/s41467-022-29999-7 (PMC9072422; doi:10.1038/s41467-022-29999-7)
Supplement: Supplementary file 3 — Reporting Summary [file 41467_2022_29999_MOESM3_ESM.pdf]

nature portfolio

Corresponding author(s): Dario Ringach

Last updated by author(s): 11/19/2021

Reporting Summary

Nature Portfolio wishes to improve the reproducibility of the work that we publish. This form provides structure for consistency and transparency in reporting. For further information on Nature Portfolio policies, see our Editorial Policies and the Editorial Policy Checklist.

Statistics

For all statistical analyses, confirm that the following items are present in the figure legend, table legend, main text, or Methods section.

n/a	Confirmed	
☐	☒	The exact sample size (*n*) for each experimental group/condition, given as a discrete number and unit of measurement
☒	☐	A statement on whether measurements were taken from distinct samples or whether the same sample was measured repeatedly
☐	☒	The statistical test(s) used AND whether they are one- or two-sided<br>*Only common tests should be described solely by name; describe more complex techniques in the Methods section.*
☒	☐	A description of all covariates tested
☒	☐	A description of any assumptions or corrections, such as tests of normality and adjustment for multiple comparisons
☐	☒	A full description of the statistical parameters including central tendency (e.g. means) or other basic estimates (e.g. regression coefficient) AND variation (e.g. standard deviation) or associated estimates of uncertainty (e.g. confidence intervals)
☐	☒	For null hypothesis testing, the test statistic (e.g. *F*, *t*, *r*) with confidence intervals, effect sizes, degrees of freedom and *P* value noted<br>*Give P values as exact values whenever suitable.*
☒	☐	For Bayesian analysis, information on the choice of priors and Markov chain Monte Carlo settings
☒	☐	For hierarchical and complex designs, identification of the appropriate level for tests and full reporting of outcomes
☒	☐	Estimates of effect sizes (e.g. Cohen's *d*, Pearson's *r*), indicating how they were calculated

Our web collection on statistics for biologists contains articles on many of the points above.

Software and code

Policy information about availability of computer code

Data collection	Scanbox (v1.13 tower)
Data analysis	Matlab (R2021b), Suite2p (v.0.7.2)

For manuscripts utilizing custom algorithms or software that are central to the research but not yet described in published literature, software must be made available to editors and reviewers. We strongly encourage code deposition in a community repository (e.g. GitHub). See the Nature Portfolio guidelines for submitting code & software for further information.

Data

Policy information about availability of data

All manuscripts must include a data availability statement. This statement should provide the following information, where applicable:

- Accession codes, unique identifiers, or web links for publicly available datasets
- A description of any restrictions on data availability
- For clinical datasets or third party data, please ensure that the statement adheres to our policy

Data and sample code are available at https://figshare.com/s/098b4ca29f5346648569

Field-specific reporting

Please select the one below that is the best fit for your research. If you are not sure, read the appropriate sections before making your selection.

☒ Life sciences ☐ Behavioural & social sciences ☐ Ecological, evolutionary & environmental sciences

For a reference copy of the document with all sections, see nature.com/documents/nr-reporting-summary-flat.pdf

Life sciences study design

All studies must disclose on these points even when the disclosure is negative.

Sample size	Sample sizes were maximized by sampling volumetric cortical areas. No prior calculation was done because, ahead of the present study there was no data that could be used to estimate the magnitude of the fluctuations in ON/OFF relative density, which would be required to calculate the number of points needed to achieve statistical power. The data collected demonstrate a statistical significant clustering at the 0.001 level in all datasets collected.
Data exclusions	Cells without significant ON or OFF maps were excluded from analysis, as they did not give reliable visual responses. Criteria for significant maps are included in the Methods.
Replication	Methods to conduct these experiments are widely available. Our analysis on the datasets collected should be replicable as we provide the databases required to do so in the Figshare link provided below. The study reports original data and did not attempt to replicate prior findings.
Randomization	Not applicable as the experimental design did not involve groups with differential treatments.
Blinding	Not applicable as the experimental design did not involve groups with differential treatments.

Reporting for specific materials, systems and methods

We require information from authors about some types of materials, experimental systems and methods used in many studies. Here, indicate whether each material, system or method listed is relevant to your study. If you are not sure if a list item applies to your research, read the appropriate section before selecting a response.

Materials & experimental systems

n/a	Involved in the study	
☒	☐	Antibodies
☒	☐	Eukaryotic cell lines
☒	☐	Palaeontology and archaeology
☐	☒	Animals and other organisms
☒	☐	Human research participants
☒	☐	Clinical data
☒	☐	Dual use research of concern

Methods

n/a	Involved in the study	
☒	☐	ChIP-seq
☒	☐	Flow cytometry
☒	☐	MRI-based neuroimaging

Animals and other organisms

Policy information about studies involving animals; ARRIVE guidelines recommended for reporting animal research

Laboratory animals	A total of 6 mice, male (2) and female (4), aged P35-56, were used. All these animals were from a cross between TRE-GCaMP6s line G6s2 (Jackson Lab, https://www.jax.org/strain/024742) and CaMKII-tTA (https://www.jax.org/strain/007004) where GCaMP6s is regulated by the tetracycline-responsive regulatory element (tetO).
Wild animals	No wild animals were used in this study
Field-collected samples	No samples from the field were collected in this study
Ethics oversight	Approved by NIH and UCLA's Office of Animal Research Oversight (the Institutional Animal Care and Use Committee)

Note that full information on the approval of the study protocol must also be provided in the manuscript.
